# Mid-term follow-up of non-ischemic heart preservation in heart transplantation

**DOI:** 10.1016/j.jhlto.2025.100285

**Published:** 2025-08-28

**Authors:** Victoria Jernryd, Oscar Braun, Audrius Paskevicius, Carsten Metzsch, Ida Haugen Lofman, Sigurdur Ragnarsson, Joanna-Maria Papageorgiou, Annika Ingvarsson, Stig Steen, Johan Nilsson

**Affiliations:** a Department of Clinical Sciences Lund, Cardiothoracic Surgery, Lund University, Lund, Sweden; b Department of Cardiothoracic and Vascular Surgery, Skane University Hospital, Lund, Sweden; c Department of Cardiology, Clinical Sciences, Lund University and Skane University Hospital, Lund, Sweden; d Division of Cardiology, Department of Medicine, Karolinska Institute, Stockholm, Sweden; e Department of Cardiology in Linköping University Hospital, Department of Health, Medicine and Caring Sciences, Linköping University, Linköping, Sweden; f Department of Translational Medicine, Thoracic surgery and bioinformatics, Lund University, Lund, Sweden

**Keywords:** Heart transplantation, Ex-vivo preservation, Survival, Primary graft dysfunction, Ischemia

## Abstract

**Background:**

Ex-vivo perfusion of donor hearts is gaining importance in minimizing ischemia-reperfusion injury during heart transplantation. The Non-Ischemic Heart Preservation (NIHP) device, developed in 2016, has shown promising results in pilot studies. This study aims to compare the mid-term follow-up outcomes of NIHP with traditional Static Cold Storage (SCS) in heart transplantation.

**Methods:**

This hybrid cohort study included 47 patients. The primary outcome was event-free survival at one year, defined as survival free of severe primary graft dysfunction (PGD), extracorporeal membrane oxygenation (ECMO) use within 7 days, acute cellular rejection (ACR ≥ 2R), and death. Secondary outcomes included graft function, incidence of adverse events at one year, and overall survival.

**Results:**

At 1 year, event-free survival was observed in 12 of 15 patients (80%) in the NIHP group and 23 of 32 patients (72%) in the SCS group. No patients in the NIHP group developed severe PGD, compared to three patients in the SCS group. ACR ≥ 2R occurred in 2/15 (13%) of NIHP patients and 5/32 (16%) of SCS patients. Overall survival at 5 years was 14/15 (93%) for NIHP and 24/32 (75%) for SCS. Immediate graft function and markers of ischemia-reperfusion injury favored the NIHP group, with lower CK-MB and lactate levels post-transplantation. Adverse events were comparable between groups, although the NIHP group had fewer severe complications.

**Conclusions:**

The NIHP system demonstrated outcomes comparable to SCS in heart transplantation, with improved graft function and reduced markers of ischemia-reperfusion injury. Further research is required to confirm these findings.

## Background

**Figure 1:**
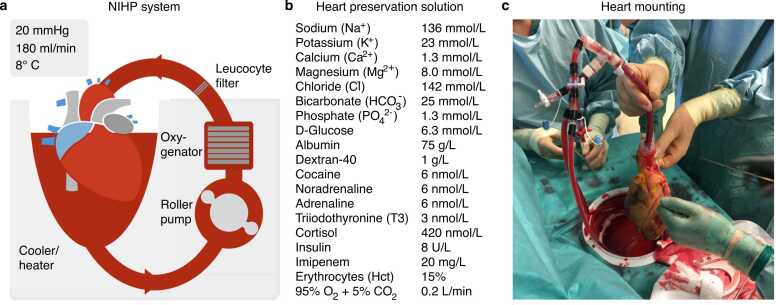
*The nonischemic heart preservation method (NIHP).* Depicts a schematic representation of the NIHP method **(a)**. The apparatus comprises a reservoir, a pressure-controlled roller pump, an oxygenator, an arterial-leucocyte filter, a cooler unit, an oxygen-and carbon dioxide container, sensors, and a programmable control system. The reservoir is filled with 2.5 L of the perfusion solution, which is then augmented by approximately 500 mL of compatible, irradiated, and leukocyte-reduced red blood cells from the hospital blood bank, resulting in a hematocrit of approximately 15% **(b)**. Perfusion is then provided through the aortic cannula to the coronary vessels. A silicon tube is placed as a vent into the left ventricle through the atrium. The heart is mounted and submerged in the heart-preservation solution, which is actively adjusted to maintain a pH of approximately 7.4. The device software is programmed to maintain a temperature of 8 °C and a mean blood pressure of 20 mmHg in the aortic root, providing a coronary flow between 150 and 250 mL/min. Figure **(c)** depicts the first human heart transplantation using the NIHP method.

Ex-vivo perfusion of donor hearts is an emerging strategy aimed at minimizing the detrimental effects of ischemia-reperfusion injury during heart transplantation.^1^ One such approach is the hypothermic cardioplegic Non-Ischemic Heart Preservation (NIHP) system, developed in 2016 by Professor Stig Steen and his team.^2^ The NIHP device provides continuous perfusion of the donor heart under hypothermic conditions (8 °C), with an oxygenated red blood cell—enriched solution under a controlled aortic root pressure (mean 20 mmHg), potentially reducing ischemic injury compared to traditional static cold storage (SCS) methods, Figure 1.

The figure is used with permission from the original publications.^3^

A preclinical study of the NIHP system enabled the first-in-human trial published in 2020, which suggested that the NIHP method might be feasible and safe for heart preservation.^3^ However, while initial results are promising, further investigation is necessary to fully assess its efficacy and safety compared to standard preservation techniques.

The development of ex-vivo perfusion techniques like NIHP provides an opportunity to extend the out-of-body preservation time safely, potentially allowing transportation from more distant donor locations. Additionally, the NIHP system may help expanding the donor pool by facilitating the use of hearts from extended criteria donors, including marginal or elderly donors, which have historically been underutilized due to their increased susceptibility to ischemia-reperfusion injury. Despite the increased risk, some of these hearts are currently used due to donor shortage with variable results.^3^, ^4^, ^5^, ^6^ Because extended criteria hearts are more susceptible to ischemia-reperfusion injury, they may benefit from preservation methods designed to reduce this injury.^1^, ^7^, ^8^, ^9^, ^10^, ^11^ By reducing ischemia-reperfusion injury, which is critical in the development of primary graft dysfunction (PGD),^12^, ^13^ ex-vivo heart perfusion may also diminish the early activation of the innate immune system.^14^, ^15^ Accordingly, a reduction of the innate immune response decreases the subsequent activation of the adaptive immune system, thereby reducing the risk of acute cellular rejection (ACR).^16^, ^17^, ^18^

However, although ex-vivo preservation techniques show potential, previous studies have not observed significant differences in early outcomes, and the long-term effects of ex-vivo perfusion on survival remain unknown.^19^, ^20^, ^21^, ^22^ This article presents the results of a one-year follow-up study comparing NIHP with SCS and examining mid-term patient survival, with the aim of improving understanding of the benefits and limitations of these cardiac preservation techniques.

## Methods

### Study design

**Figure 2:**
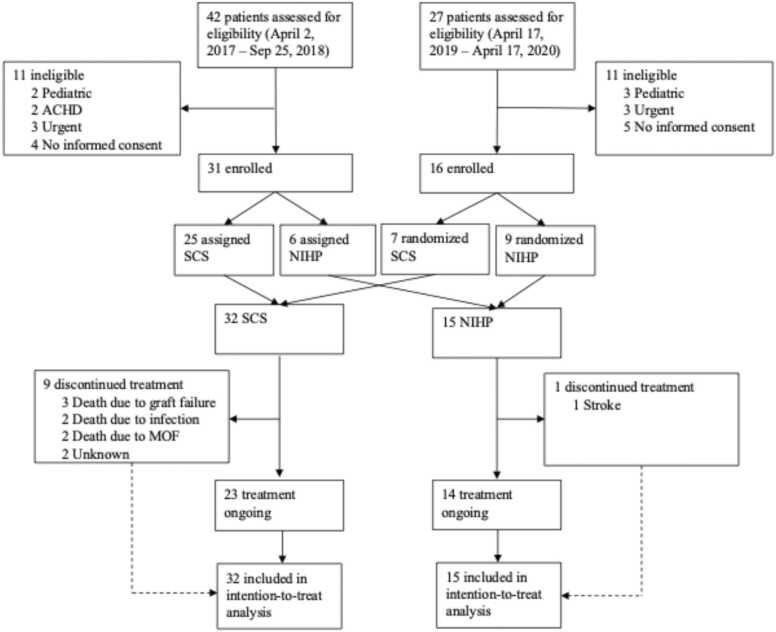
*CONSORT flow diagram.* shows a modified CONSORT flow diagram for all recipients enrolled in the trial. *MOF* multi-organ failure, *ACHD* adult congenital heart disease, *NIHP* non-ischemic heart preservation, and *SCS* static cold storage.

This study utilized a hybrid cohort integrating data from two parts of a clinical trial: a non-randomized prospective component and a subsequent randomized component [ClinicalTrial.gov, number NCT03150147].^3^ The first cohort consisted of 31 patients from the previously conducted non-randomized prospective study. Subsequently, 16 additional patients were recruited from the randomized study, following the same protocol as the first cohort, Figure 2. The primary objective was to evaluate outcomes at the one-year follow-up using a consistent protocol throughout. The study received approval from the Ethics Committee in 2016 (2016/603) and was conducted in accordance with the Declaration of Helsinki.

### Patient eligibility and consent

Inclusion criteria for the recipients were 18 years or older, no congenital heart disease, and normal kidney function. Inclusion criteria for the donors were 70 years or younger, brain dead, no clinically meaningful coronary artery disease, and normal left ventricular ejection fraction (LVEF). Written informed consent was obtained from all patients prior to their inclusion in the study. Detailed criteria are presented in Supplementary Material and Methods.

### Transplantation procedures for the total cohort

The transplantation process was coordinated urgently for all patients. The total preservation time was defined as the donor heart’s out-of-body time (ie, cross-clamp on the donor aorta at the donor hospital until release of cross-clamp in the recipient at the transplant center). Upon arrival at the transplantation hospital, cold blood cardioplegia was administered from the heart-lung machine and readministered after each anastomosis. The time from the recipient cross-clamp to reperfusion is referred to as implantation time. Cold ischemia time refers to the length of time that the donor heart was kept cold without any continuous perfusion.

### Heart preservation

The NIHP device operates as a miniaturized heart-lung machine housed in a portable box. It comprises a reservoir, a pressure-controlled roller pump, an oxygenator, an arterial-leucocyte filter, a cooler unit, and a programmable control system. It utilizes a priming volume of 3 L with a special cardioplegic solution (Heart Solution with Heart Solution Supplement, XVIVO group, Gothenburg, Sweden) containing two units of packed erythrocytes, O Rh negative, from the blood bank. The system maintains a temperature of 8 °C and a mean blood pressure of 20 mmHg in the aortic root. The oxygenator is subjected to a constant gas flow of 0.2 L per minute, comprising 95% oxygen and 5% carbon dioxide. These gases are drawn from an oxygen-carbon dioxide container. The donor heart was arrested using the same preservation solution devoid of erythrocytes, and perfusion was initiated immediately after cannulation and connection to the NIHP system, Figure 1.

In the SCS method, the donor heart is arrested using a cold crystalloid cardioplegic solution (Plegisol, Pfizer). Subsequently, the heart is placed in a sterile bag containing 1,000 mL of the approximately 4 °C cardioplegic solution. This bag is then placed in a second bag containing 1,000 mL of cold solution and finally placed in a third bag. This pack is then placed in the cool box with a rubber barrier between the ice and the heart. This packaging technique prevents direct contact between the heart and the ice, thus preventing freeze damage.

### Study outcomes and measurements

#### Primary outcome

Event-Free Survival During the First Year: The primary outcome was defined as survival free of severe PGD at 24 hours, extracorporeal membrane oxygenation (ECMO) use within 7 days, ACR ≥ 2R, and death at 1 year after transplantation. PGD was graded according to the International Society of Heart and Lung Transplantation (ISHLT) guidelines^12^ and the grading of ACR was made blinded to the study groups by pathologists as a part of routine care according to the ISHLT guidelines.^23^

#### Secondary outcomes

##### Mid-term survival

Overall survival from any cause up to seven years post-transplantation was measured. The latest follow-up occurred on April 14, 2024.

##### One-year follow-up assessments

Evaluation of graft function using echocardiography; Measurement of cardiac index, mean arterial pressure, mean pulmonary artery pressure, wedge pressure, right atrial pressure, and pulmonary vascular resistance through right heart catheterization; Assessment of coronary stenosis and coronary artery vasculopathy via coronary angiography; Collection of biomarkers such as creatinine, bilirubin, urea, NT-proBNP, and hemoglobin to evaluate renal and liver function and the degree of heart failure; measurement of tacrolimus concentrations to determine the required dose of immunosuppression.

##### Short-term postoperative measurements

Cardiac index at 6 ± 2 and 24 ± 6 hours after the end of preservation; Grading of PGD; Creatine kinase-muscle/brain (CK-MB) and lactate at 6 ± 2 and 24 ± 6 hours after preservation (Triage CARDIO3, Alere with Biosite TriageMeterPro; Blood gas analyzer Radiometer, Copenhagen, Denmark); Peak creatinine within 24 hours and the need for continuous renal replacement therapy (CRRT) within 7 days post-transplantation; Peak aspartate aminotransferase (AST) and alanine transaminase (ALT) within 24 hours post-transplantation.

##### Adverse events

Acute Cardiac-Related Events; Acute Respiratory Failure; Acute Kidney Failure; Acute Liver Failure; Permanent stroke; and Permanent pacemaker. Detailed definitions are presented in Supplementary Material and Methods.

### Statistical analysis

The primary outcome, event-free survival, was analyzed using the Kaplan-Meier method. The Kaplan-Meier estimate is presented with 95% CIs. For patients who had more than one event during follow-up that resulted in failure to reach the primary endpoint, the event that occurred first is the one included in the analysis. Heart size in grams for recipients and donors was calculated using an established equation for predicted heart mass (pHM).^24^ Continuous variables were summarized as medians with interquartile ranges (IQR) for non-normally distributed data or mean with standard deviations for normally distributed data. Categorical variables were summarized as frequencies and percentages. Because of the small sample size in both groups, only descriptive statistics were performed. Statistical analyses were performed using Stata MP statistical package, version 15.1 (Revision 03 Feb 2020, StataCorp LP, College Station, TX).

## Results

### Recruitment

Between April 2017 and April 2020, 69 patients assessed for eligibility (Figure 2). Of these, 22 patients were excluded, seven met one of the exclusion criteria, four did not provide written informed consent, five were unable to provide consent due to a short time on the waiting list, and six required urgent heart transplantation. In total, 47 patients were included in the study, where the NIHP system was assigned to 15 patients and SCS to 32 patients (Figure 2). Following organ retrieval, all organs were utilized, and all patients were followed up for at least 12 months with no missing data on outcomes, resulting in a median follow-up time of 68 months (IQR, 50-74).

### Patient and donor characteristics

The characteristics of donors and recipients in the two study groups are shown in Table 1. Overall, 12/47 (26%) recipients and 14/47 (30%) donors were women. The median age was 55 years (IQR, 44-63) for recipients and 51 years (IQR, 35-58) for donors. Baseline characteristics were similar for those in the two groups. The pHM recipient/donor ratio was 1.03 ± 0.11 in NIHP, compared to 0.95 ± 0.10 in the SCS group. The out-of-body time was longer with NIHP, with a median of 248 min (IQR, 204-285) compared to 198 min (IQR, 158-226) with SCS. The implantation time, which is a part of the out-of-body time, was longer in the NIHP group, 110 min (IQR, 94-130) compared to 87 min (IQR, 80-102) in the SCS group.

**Table 1:** Donor, Recipient, and Transplantation Characteristics (*n* = 47)

	*n*	NIHP *n* = 15	SCS *n* = 32
*Donor characteristics*			
Age	47	48 (39-59)	52 (32-58)
Female sex	47	3 (20%)	11 (34%)
BMI (kg/m^2^)	47	25.9 ± 3.6	27.4 ± 6
Blood group	47		
O		5 (33%)	16 (50%)
A		9 (60%)	8 (25%)
AB		0	3 (9%)
B		1 (7%)	5 (16%)
History of smoking	41	8 (53%)	10 (32%)
Hypertension	45	2 (13%)	11 (37%)
CMV	47	11 (73%)	25 (78%)
*Recipient characteristics*			
Age	47	56 (43-64)	54 (46-63)
Female sex	47	2 (13%)	10 (31%)
BMI (kg/m^2^)	47	26.7 ± 3.6	25.6 ± 4.2
Diagnosis	47		
Ischemic Cardiomyopathy		3 (20%)	9 (28%)
Non-Ischemic Cardiomyopathy		10 (67%)	20 (63%)
Other		2 (13%)	3 (9%)
Blood group	47		
O		4 (27%)	13 (41%)
A		10 (67%)	9 (28%)
AB		0	4 (13%)
B		1 (7%)	6 (19%)
Diabetes, insulin-treated	47	1 (7%)	4 (13%)
Peripheral vascular disease	46	1 (7%)	6 (19%)
History of stroke	43	2 (14%)	1 (3%)
Creatinine (μmol/L)	47	108 (91-134)	94 (82-116)
Bilirubin (μmol/L)	47	11 (9-28)	12 (7-20)
CMV	47	9 (60%)	23 (72%)
PVR (Wood units)	45	2.06 ± 0.59	2.07 ± 0.7
Panel Reactive Antibodies	43		
0%-10%		6 (40%)	10 (31%)
11%-80%		6 (40%)	12 (38%)
>80%		2 (13%)	7 (22%)
LVAD	47	7 (47%)	16 (50%)
*Transplantation details*			
Volume of cardioplegia (mL)	47	1400 (1,300-1,800)	1900 (1,800-2,000)
Out of body time (min)	47	248 (204-285)	198 (158-226)
CPB (min)	47	208 (177-239)	181 (164-213)
Implantation time (min)	47	110 (94-130)	87 (80-102)
Recipient/Donor pHM ratio	47	1.03 ± 0.11	0.95 ± 0.10
Female donor to male recipient	47	2 (13%)	1 (3%)

Data are *n* (%), median (IQR) or mean (±SD). BMI, body mass index; CMV, cytomegalo virus; CPB, cardiopulmonary bypass; IQR, interquartile range; LVAD, left ventricular assist device; *n*, numbers; NIHP, nonischemic heart preservation; pHM, predicted heart mass; PVR, pulmonary vascular resistance; ratio, a ratio >1 represent heavier/larger recipients compared to donors, a ratio <1 represent heavier/larger donors compared to recipients; SCS, static cold storage; SD, standard deviation.

### Ex-vivo perfusion data

For the NIHP group, the median cold ischemic time before the start of NIHP was 18 min (IQR 16-25, range 8-30). The heart was then perfused for a median of 162 min (IQR 109-184, range 26-218) with a coronary blood flow of 184 mL/min (IQR, 158-221, range 143-313). The pre-programmed settings of 20 mmHg pressure in the aortic root and 8 °C temperature were stable, and all NIHP procedures were uneventful. Lactate levels at the end of preservation were 1.5 mmol/L (IQR 1.3-1.6, range 1.0-1.7).

### Primary outcome

**Figure 3:**
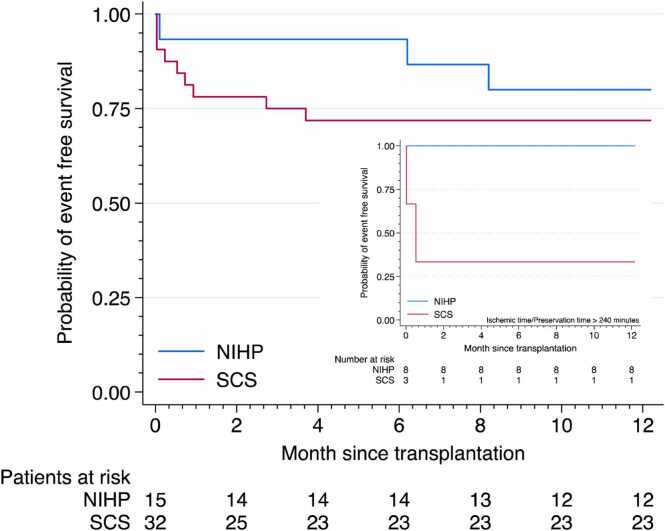
*Probability of event-free survival during the first year after heart transplantation.* The Kaplan-Meier plot shows the probability of event-free survival (primary composite endpoint), defined as survival free of severe primary graft dysfunction at 24 hours, free of extracorporeal mechanical support use at 7 days, and free of acute cellular rejection ≥ 2R and death at one year (cyan: NIHP group; red: SCS group). The Kaplan-Meier estimated free of event was 80% (95% CI 50-93) for the NIHP group and 72% (95% CI 53-84) for the SCS group. The embedded figure shows the probability of event-free survival for a subgroup of transplantations with a preservation time of more than 4 hours.

In the first year, the primary endpoint, defined as event-free survival of PGD, ECMO within seven days, ACR ≥ 2R, and death, was achieved by 12/15 patients in the NIHP group (Table 2), resulting in a Kaplan-Meier estimate of event-free survival of 80% (95% confidence interval [CI], 50%-93%), Figure 3. In the SCS group, 23/32 patients met the primary endpoint, with an event-free survival estimate of 72% (95% CI, 53%-84%). Among those who received a graft with an out-of-body time exceeding four hours (*n* = 11), all had an event-free one-year survival in the NIHP group (*n* = 8) while one patient in the SCS group had a non-fatal PGD with ECMO support and one with non-fatal ACR 2R (*n* = 3), Figure 3.

**Table 2:** Primary Outcome and Outcomes at 1 Year After Heart Transplantation (*n* = 47)

		*n*	NIHP *n* = 15	SCS *n* = 32
*Primary outcome*				
Survival free of event within the first year		47	12 (80%)	23 (72%)
*First event that resulted in failure to reach the primary outcome*				
Severe PGD within 24 hours		47	0	3 (9%)
ECMO within 7 days		47	0	1 (3%)
ACR ≥ 2R within the first year		47	2 (13%)	4 (13%)
Death within the first year		47	1 (7%)	1 (3%)

ACR, acute cellular rejection; ECMO, extra corporeal membrane oxygenation; *n*, numbers; NIHP, nonischemic heart preservation; PGD, primary graft dysfunction; SCS, static cold storage.

In a separate sub-analysis of the individual components of the composite outcome variable, none of the patients in the NIHP group developed severe PGD, whereas three patients (9%) in the SCS group did, of which two died within three months after transplantation. Additionally, one patient in the SCS group required ECMO on day seven due to cardiac dysfunction. Within the first year, ACR 2R occurred in two patients 2/15 (13%) in the NIHP group and five patients 5/32 (16%) in the SCS group. No patient had any ACR 3R. In the SCS group 5/32 (16%) patients died during the first year after transplantation, while in the NIHP group, 1/15 (7%) died in the first year; the latter was an initial postoperative fatality due to massive stroke.

**Figure 4:**
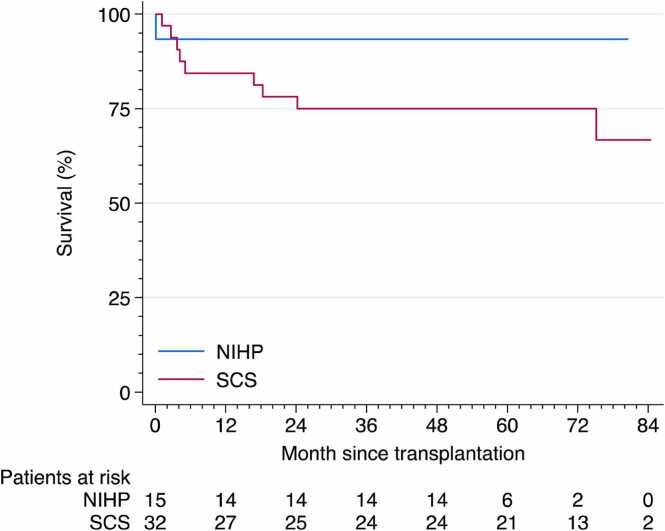
*Overall survival after heart transplantation.* Shows the Kaplan-Meier plot for the overall survival after heart transplantation (cyan: NIHP group; red: SCS group). The Y-axis displays survival (%), and the X-axis represents time since transplantation. The estimated 5-year overall survival was 93% (95% CI, 61%-99%) for the NIHP group and 75% (95% CI, 56%-87%) for the SCS group.

After the 1-year follow-up, four additional deaths were observed in the SCS group, yielding a 5-year Kaplan–Meier overall survival estimate of 75% (95% CI, 56%-87%) for the SCS group compared with 93% (95% CI, 61%-99%) for the NIHP group, Figure 4.

### One-year follow-up assessment

At the 1-year follow-up (Table 3), right heart catheterization showed no differences between the NIHP and SCS groups in cardiac index, mean arterial pressure, mean pulmonary artery pressure, pulmonary artery wedge pressure, right atrial pressure, and pulmonary vascular resistance. Echocardiography revealed normal left ventricular ejection fraction (>50%) in 13/14 (93%) of NIHP patients and 23/27 (85%) of SCS patients and a normal right ventricular ejection fraction in 6/14 (43%) of NIHP patients and 15/27 (58%) of SCS patients. Coronary angiography identified one SCS patient with significant stenosis. There was one patient on everolimus treatment in the NIHP group and two patients in the SCS group. All other patients were treated with tacrolimus. Laboratory samples indicated no significant differences in creatinine, bilirubin, urea, NT-proBNP, hemoglobin, or tacrolimus concentration between the groups.

**Table 3:** One Year Follow-Up (*n* = 41)

	*n*	NIHP *n* = 14	SCS *n* = 27
*Right heart catheterization*			
Cardiac Index (L/min/m^2^)	37	2.82 ± 0.52	2.75 ± 0.42
Mean Arterial Pressure (mmHg)	32	96 (91-110)	94 (89-110)
Mean Pulmonary Artery Pressure (mmHg)	39	19 ± 6	16 ± 5
Pulmonary Artery Wedge Pressure (mmHg)	39	10 ± 4	8 ± 4
Right Atrial Pressure (mmHg)	39	4.33 ± 2.87	3.63 ± 2.68
PVR (Wood units)	39	1.52 ± 0.55	1.57 ± 0.67
*Echocardiography*			
Left Ventricle Ejection Fraction	41		
Normal >50%		13 (93%)	23 (85%)
Mildly reduced 40%-50%		1 (7%)	2 (7%)
Moderately reduced 30%-40%		0	2 (7%)
Right Ventricle Ejection Fraction	40		
Normal >50%		6 (43%)	15 (58%)
Slightly reduced 40%-50%		8 (57%)	9 (35%)
Moderately reduced 30%-40%		0	2 (8%)
*Coronary angiography*			
Significant stenosis	39	0	1 (3%)
Cardiac Allograft Vasculopathy grade 1	36	1 (7%)	2 (6%)
*Laboratory samples*			
Creatinine (μmol/L)	41	100 (87-127)	93 (86-113)
Urea (mmol/L)	39	9 ± 5.9	8 ± 3.3
NT-proBNP (ng/L)	39	329 (237-682)	323 (219-858)
Tacrolimus concentration (ng/mL)	39	6.6 ± 1.1	7.4 ± 1.8

*n*, numbers; NIHP, nonischemic heart preservation; NTproBNP, N-terminal pro b-type natriuretic peptide; PGD, primary graft dysfunction; PVR, pulmonary vascular resistance; SCS, static cold storage.

### Short-term postoperative measurements

Postoperative assessments for immediate graft function are presented in Table 4. In the NIHP group, six out of 15 patients (40%) were diagnosed with mild left-sided PGD, while the remaining nine patients (60%) showed no PGD. In contrast, in the SCS group only 6 of 32 patients (19%) had no PGD, whereas 20 of 32 (63%) had mild PGD, 3 of 32 (9%) had moderate PGD, and 3 of 32 patients (9%) experienced severe PGD, all of whom required postoperative VA-ECMO support, *p* = 0.041. Right-sided PGD was present in three patients (9%) in the SCS group but was absent in the NIHP group. The cardiac index measured at 24 ± 6 hours postoperatively was higher in the NIHP group compared to the SCS group (3.3 ± 0.6 L/min/m² vs 2.8 ± 0.6 L/min/m²; *p* = 0.030). Markers of ischemia-reperfusion injury were lower in the NIHP group at 6 ± 2 hours, with CK-MB levels of 77 ng/mL (IQR, 51-144) compared to 137 ng/mL (IQR, 73-181; *p* = 0.031) in the SCS group. Furthermore, lactate levels were lower in the NIHP group, with a median of 2.0 mmol/L (IQR, 1.6-4.9) versus 4.6 mmol/L (IQR, 3.1-8.6; *p* = 0.030) in the SCS group. Renal function assessment showed higher creatinine levels at 24 ± 6 hours in the NIHP group, 212 µmol/L (IQR, 120-233), compared to 154 µmol/L (IQR, 101-186) in the SCS group. The incidence of CRRT within 7 days was also higher in the NIHP group, nine out of 15 patients (60%), compared to the SCS group, 12 out of 32 patients (38%). Liver function, measured by AST levels at 24 ± 6 hours, was lower in the NIHP group 1.6 µkat/L (IQR, 1.3-2.5) compared to 2.6 µkat/L (IQR, 2.2-3.8) in the SCS group, while ALT levels were similar between the groups.

**Table 4:** Secondary Outcomes (*n* = 47)

	*n*	NIHP *n* = 15	SCS *n* = 32
*Immediate graft function*			
PGD (left)	47		
No		9 (60%)	6 (19%)
Mild		6 (40%)	20 (63%)
Moderate		0	3 (9%)
Severe		0	3 (9%)
PGD (right)	47	0	3 (9%)
Cardiac index 6 ± 2 hours (L/min/m^2^)	36	3.0 ± 0.5	2.8 ± 0.7
Cardiac index 24 ± 6 hours (L/min/m^2^)	38	3.3 ± 0.6	2.8 ± 0.6
*Ischemia-Reperfusion Injury*			
CK-MB 6 ± 2 hours (ng/mL)	46	77 (51-144)	137 (73-181)
CK-MB 24 ± 6 hours (ng/mL)	44	29 (16-47)	24 (12-50)
Lactate 6 ± 2 hours (mmol/L)	47	2.0 (1.6-4.9)	4.6 (3.1-8.6)
Lactate 24 ± 6 hours (mmol/L)	47	2.4 (2-3.6)	2.9 (2.4-4.1)
*Renal function*			
Creatinine 24 ± 6 hours (μmol/L)	46	212 (120-233)	154 (101-186)
CRRT within 7 days	47	9 (60%)	12 (38%)
*Liver function*			
AST 24 ± 6 hours (μkat/L)	46	1.6 (1.3-2.5)	2.6 (2.2-3.8)
ALT 24 ± 6 hours (μkat/L)	46	0.5 (0.4-0.6)	0.5 (0.4-0.8)

ALT, alanine transaminase; AST, aspartate aminotransferase; CK-MB, creatinine kinase-muscle/brain; CRRT, continuous renal replacement therapy; *n*, numbers; NIHP, nonischemic heart preservation; PGD, primary graft dysfunction; SCS, static cold storage.

### Adverse events

In the NIHP group, there were 17 (1.1 per patient) non-fatal serious adverse events (the fatalities were as noted previously), including respiratory failure in 4/15 (27%) patients, acute kidney failure in 11/15 (73%) patients, and permanent stroke in 2/15 (13%) patients. No cases of acute cardiac failure, acute bleeding, acute liver failure, or the need for a permanent pacemaker were observed. In the SCS group, there were 43 (1.3 per patient) non-fatal serious adverse events, including acute cardiac failure in 4/32 (13%) patients, acute bleeding in 2/32 (6%) patients, respiratory failure in 11/32 (34%) patients, acute kidney failure in 20/32 (63%) patients, acute liver failure in 1/32 (3%) patients, permanent stroke in 1/32 (3%) patients, and the need for a permanent pacemaker in 4/32 (13%) patients 13%.

## Discussion

This mid-term follow-up of NIHP in heart transplantation strengthens the evidence for its efficacy and safety. Our findings suggest that NIHP offers comparable outcomes in terms of event-free survival, graft function, and postoperative complications when compared to the conventional SCS method.

In our study, event-free survival at 1 year was observed in 80% (12/15) of patients in the NIHP group, compared to 72% (23/32) in the SCS group. Furthermore, none of the patients in the NIHP group developed moderate or severe PGD, whereas six patients in the SCS group did, with three requiring ECMO support. The cardiac index and freedom from PGD at 24 hours were better in the NIHP group. These results align with the preliminary findings of the European study (NIHP2019), which reported a significant reduction in PGD rates in patients who received grafts preserved with NIHP method.^25^, ^26^ PGD is a serious complication that can lead to death in heart transplantation,^12^, ^13^, ^27^, ^28^ and reducing its incidence is a critical goal.

The NIHP group exhibited lower postoperative levels of CK-MB and lactate compared to the SCS group, suggesting reduced ischemia-reperfusion injury and improved circulation. While these biomarkers have limitations, they are valuable in donor heart evaluation. Previous studies have associated lower CK-MB and lactate levels with reduced risk of PGD and better hemodynamic stability in heart transplantation.^29^, ^30^, ^31^ The lower values in the NIHP group may reflect a reduction in ischemia-reperfusion injury, which could decrease the innate immune response and the risk of PGD and ACR.^15^, ^16^, ^17^, ^18^ However, no differences in ACR between the groups were detected in this study. The outcome of transplantation is multifactorial, involving complex immune responses that are not yet fully understood. Larger studies on NIHP are required to fully evaluate its influence on the immune system.

The preservation time with NIHP was longer compared to SCS, which was expected due to the necessary preparation and cannulation before perfusion. The NIHP group also exhibited a longer implantation time, which may reflect more complex surgical procedures or a reduced sense of urgency due to the continuous perfusion of the donor heart. Among patients with out-of-body times exceeding four hours, all eight in the NIHP group had event-free 1-year survival, compared to only one of the three patients in the SCS group with prolonged ischemic time (Figure 3, inset). Although based on a limited number of patients, this observation is consistent with findings from McGiffin et al.,^32^ who reported favorable outcomes with preservation times up to nearly nine hours. Additionally, the first transatlantic NIHP on a commercial flight was recently conducted with a preservation time of 12 hours.^33^ These findings suggest that NIHP may extend out-of-body times compared to SCS; however, other techniques, such as the TransMedics Organ Care System, are also available and have been used to extend preservation times.^19^, ^34^ Further studies are needed to compare these different preservation methods directly and to place our findings within the broader context of alternative beating and non-beating preservation and transport systems.

Adverse events such as acute renal failure and respiratory failure were comparable between the two groups. Although the NIHP group showed higher creatinine level 24 hours post-transplant, renal function had normalized in both groups by the 1-year follow-up.

The NIHP system functioned properly for all hearts without the need for adjustments after the start of perfusion, indicating that the method is reliable and manageable by a typical donor harvesting team. The simplicity of NIHP is a strength, and the hypothermic, cardioplegic state of the heart provides an added safety margin in case of technical issues. While other systems, such as the TransMedics Organ Care System, preserve the heart in a nearly physiological state and have their own advantages, they may require more specialized training.^19^, ^34^ Further comparative studies are necessary to evaluate the relative benefits and limitations of these different preservation methods.

This study has several limitations. Because it was a non-randomized trial, bias in the selection of both donors and recipients could have affected the results. The sample size was small, limiting the statistical power to detect significant differences between the groups. Additionally, the study was conducted at a single center, which may affect the generalizability of the findings. Moreover, the temperature in SCS was not measured, and therefore the influence of temperature on the result cannot be analyzed.

In conclusion, NIHP appears to be a promising alternative to SCS for heart transplantation, offering comparable outcomes in terms of event-free survival, graft function, and postoperative complications. This study provides pilot data that justify further investigation into the potential benefits of NIHP in heart transplantation.

## Disclosure statement

The study was supported by the Swedish Research Council (2019-00487 and 2023-03184), Vinnova (2017-04689), Swedish Heart-Lung Foundation (20190623 and 20220225), a government grant for clinical research, region Skane research funds, donation funds from Skane University Hospital, the Anna-Lisa and Sven Eric Lundgrens Foundation, Hans-Gabriel and Alice Trolle-Wachmeister’s Foundation for Medical Research, and Hjelms Family Foundation for Medical Research. The supporting sources were not involved in the study.

## Author contributions

VJ: study design, patient coordination, personnel education, data interpretation, data analysis, and the writing of the manuscript. OB: data interpretation, analysis and collection, and final manuscript review. AP: participation in the donor organ explantation, responsible for the programming of the NIHP system, and data collection. CM: data interpretation, analysis and collection, and final manuscript review. IHL: data interpretation, analysis and collection, and final manuscript review. SR: personnel education and participation in the organ harvesting, data collection, and review of the final manuscript. JMP: data interpretation, analysis and collection, and final manuscript review. AI: data interpretation, analysis and collection, and final manuscript review. SS: study design, device invention, and final manuscript review. JN: the role of the principal investigator, study design, data interpretation, data analysis, design of the infographic illustrating the NIHP method, personnel education, participation in organ harvesting and transplantation, and review of the final manuscript.

## Data Availability

Due to legal and ethical restrictions regarding patient confidentiality, raw data cannot be shared publicly. However, interested researchers can request access to the raw data by contacting the corresponding author, J.N. Instructions on how to apply and the criteria for accessing confidential data are available on the Swedish Ethical Review Authority website (http://etikprovning.se).

## Declaration of Competing Interest

The authors declare that they have no known competing financial interests or personal relationships that could have appeared to influence the work reported in this paper.

## Footnotes

Appendix A Supplementary data associated with this article can be found in the online version at doi:10.1016/j.jhlto.2025.100285.
