# Supplementary material for: Mid-term follow-up of non-ischemic heart preservation in heart transplantation
Source: JHLT Open. 2025 Aug 28;9:100285. doi: 10.1016/j.jhlto.2025.100285 (PMC12861730; doi:10.1016/j.jhlto.2025.100285)
Supplement: Supplementary file 1 — Supplementary material [file mmc1.docx]

**Supplementary Information for**

**Mid-term follow-up of Non-Ischemic Heart Preservation in heart transplantation**

Victoria Jernryd, Oscar Braun, Audrius Paskevicius, Carsten Metzsch, Ida Haugen Lofman, Sigurdur Ragnarsson, Joanna-Maria Papageorgiou, Annika Ingvarsson, Stig Steen, Johan Nilsson.

**Materials and Methods**

**Patient Eligibility and Consent**

Organ donors had to be brain dead, 70 years or younger. Donors were excluded if any of the following criteria were fulfilled: insulin-treated diabetes, significant coronary artery disease, hepatitis B- B-positive or hepatitis C-positive serology, human immunodeficiency virus-positive serology, tuberculosis, malignancy, and abnormal ventricular function <45%.

All adult (aged 18 years or older) recipients on our waiting list for heart transplantation were eligible; however, we excluded those who previously underwent solid organ or bone marrow transplantation, had grown up congenital heart disease, had undergone four or more sternotomies, had known malignancy, had kidney failure (Iohexol plasma clearance <30 at listing), had liver failure (aspartate aminotransferase, alanine transaminase, or total bilirubin more than five times the upper limit of normal, or international normalized ratio >2.0), had ongoing septicemia, and had urgent, and/or systemic inflammatory disorders treated with corticosteroids. ﻿

Potential participants provided consent while on the waiting list, and that consent was affirmed on the day of transplantation. Consent included allowing the recording of anonymized data for trial purposes and the collection of biological samples for storage in the trial biobank.

**Study Outcomes and Measurements**

Event-Free Survival During the First Year:

- The primary outcome was event-free survival during the first year following transplantation. Event-free survival is defined as the time from randomization to the occurrence of any adverse event, including disease progression, recurrence, or death from any cause.

Long-Term Survival:

- Overall survival up to seven years post-transplantation was measured, evaluating overall survival from the time of randomization until death from any cause.

One-Year Follow-Up Assessments:

- Graft and Patient Survival: Survival rates of both the graft and the patient at the one-year mark.
- Acute Cellular Rejection: Incidence of ACR during the first year after transplantation.
- Graft Function: Evaluation of graft function by measuring LVEF and RVEF using echocardiography.
- Hemodynamic Parameters: Measurement of cardiac index, mean arterial pressure, mean pulmonary artery pressure, wedge pressure, right atrial pressure, and pulmonary vascular resistance through right heart catheterization.
- Coronary Health: Assessment of coronary stenosis and coronary artery vasculopathy via coronary angiography.
- Biomarker Analysis: Collection of biomarkers such as creatinine, bilirubin, urea, NT-proBNP, and hemoglobin to evaluate renal and liver function, and the degree of heart failure. Tacrolimus concentrations were also measured to determine the required dose of immunosuppression.

Short-Term Postoperative Measurements:

- Immediate Graft Function: Assessment of cardiac index at 6±2 and 24±6 hours after the end of preservation.
- Primary Graft Dysfunction: Grading of PGD based on LVEF <40% and RVEF <40% with echocardiography and hemodynamic measurements on the first postoperative day.
- Ischemia-Reperfusion Injury: Measurement of creatine kinase-muscle/brain (CK-MB) and lactate levels at 6±2 and 24±6 hours after preservation.
- Renal Function: Evaluation of peak creatinine levels within 24 hours and the need for continuous renal replacement therapy (CRRT) within 7 days post-transplantation.
- Liver Function: Assessment of peak aspartate aminotransferase (ASAT) and peak alanine transaminase (ALAT) levels within 24 hours post-transplantation.

Adverse Events

- Acute Cardiac-Related Events: Defined as the need for an intra-aortic balloon pump and/or mechanical circulatory support within 7 days post-transplantation.
- Acute Bleeding: Defined according to the Bleeding Academic Research Consortium (BARC) type IV criteria (>2000 mL/24 hours and/or requiring re-operation for bleeding, intracranial bleeding, and/or transfusion of >5 red blood cell concentrates/48 hours).
- Respiratory Failure: Defined as impairment of respiratory function requiring re-intubation, tracheostomy, or the inability to discontinue invasive ventilator support within 48 hours after cardiopulmonary bypass due to respiratory issues rather than sedation.
- Acute Kidney Failure: Defined according to the Kidney Disease Improving Global Outcomes (KDIGO) criteria as an increase in serum creatinine of >27 μmol/L within 48 hours or 1.5 times baseline within 7 days.
- Acute Liver Failure: Defined as the rapid development of hepatocellular dysfunction, specifically coagulopathy and mental status changes (encephalopathy) in a patient without prior known liver disease.
- Permanent Stroke: Defined as an episode of computed tomography (CT)-verified acute neurological dysfunction caused by ischemia or hemorrhage persisting ≥24 hours or until death.
- Permanent Pacemaker Requirement: Defined as the need for a permanent pacemaker implantation two weeks after transplantation.
